# Access to Basic HIV-Related Services and PrEP Acceptability among Men Who Have sex with Men Worldwide: Barriers, Facilitators, and Implications for Combination Prevention

**DOI:** 10.1155/2013/953123

**Published:** 2013-07-08

**Authors:** George Ayala, Keletso Makofane, Glenn-Milo Santos, Jack Beck, Tri D. Do, Pato Hebert, Patrick A. Wilson, Thomas Pyun, Sonya Arreola

**Affiliations:** ^1^The Global Forum on MSM & HIV (MSMGF), 436 14th Street, Suite 1500, Oakland, CA 94612, USA; ^2^Department of Epidemiology and Biostatistics, University of California, San Francisco, San Francisco, CA, USA; ^3^San Francisco Department of Public Health, San Francisco, CA, USA; ^4^Center for AIDS Prevention Studies, University of California, San Francisco, San Francisco, CA, USA; ^5^Mailman School of Public Health, Columbia University, New York, NY, USA; ^6^Urban Health Program, RTI International, San Francisco, CA, USA

## Abstract

*Introduction*. Men who have sex with men (MSM) are disproportionately impacted by HIV globally. Easily accessible combination HIV prevention strategies, tailored to the needs of MSM, are needed to effectively address the AIDS pandemic. *Methods and Materials*. We conducted a cross-sectional study among MSM (*n* = 3748) from 145 countries from April to August 2012. Using multivariable random effects models, we examined factors associated with acceptability of preexposure prophylaxis (PrEP) and access to condoms, lubricants, HIV testing, and HIV treatment. *Results*. Condoms and lubricants were accessible to 35% and 22% of all respondents, respectively. HIV testing was accessible to 35% of HIV-negative respondents. Forty-three percent of all HIV-positive respondents reported that antiretroviral therapy was easily accessible. Homophobia, outness, and service provider stigma were significantly associated with reduced access to services. Conversely, community engagement, connection to gay community, and comfort with service providers were associated with increased access. PrEP acceptability was associated with lower PrEP-related stigma, less knowledge about PrEP, less outness, higher service provider stigma, and having experienced violence for being MSM. *Conclusions*. Ensuring HIV service access among MSM will be critical in maximizing the potential effectiveness of combination approaches, especially given the interdependence of both basic and newer interventions like PrEP. Barriers and facilitators of HIV service access for MSM should be better understood and addressed.

## 1. Introduction

HIV surveillance studies show that men who have sex with men (MSM) continue to shoulder a disproportionate HIV disease burden compared with the general population in virtually every country for which there is reliable surveillance data [1]. This fact has been true since the epidemic began in the early 1980s [2]. 

In many high-income countries, incidence of HIV among MSM continues to climb even while overall HIV incidence is in decline. In the United States, the number of new HIV infections among MSM has been increasing at a rate of 8% per year since 2001 [3, 4]. HIV prevalence across North, South, and Central America, South and Southeast Asia, and Sub-Saharan African ranges consistently between 14 and 18% [2].

Due to stigma, discrimination, and criminalization, the HIV epidemic among MSM continues to go largely unaddressed in many parts of the world. As of December 2011, 93 countries had failed to report any data on HIV prevalence among MSM over the previous 5 years [5], and recent reports indicate that less than 2% of global HIV prevention funding is directed toward MSM [6].

These troubling trends are taking place against the backdrop of a shifting HIV prevention and treatment landscape. Randomized controlled trials have shown the prevention potential of biomedical interventions like preexposure prophylaxis (PrEP) among MSM and early initiation of antiretroviral treatment to prevent forward transmission between serodiscordant heterosexual couples [7, 8]. Trial findings are consistent with observational and ecologic studies that have noted the association between HIV treatment and reductions in new HIV infections [9, 10]. 

Recent advances underscore the need to develop and implement carefully planned combination prevention approaches tailored to the needs and concerns of MSM in a wide range of contexts to reduce new HIV infections in this population [11–14]. However, if MSM are to benefit from approaches that combine new and existing biomedical, behavioral, and structural interventions, factors that impact access to and acceptability of these interventions for MSM must be clearly described and addressed. 

In 2012, the Global Forum on MSM and HIV (MSMGF) developed and implemented the Global Men's Health and Rights Survey (GMHR), an international multilingual online questionnaire designed to identify and examine barriers and facilitators that affect HIV service access for MSM around the world. We were particularly interested in understanding access to and acceptability of various service components that could comprise combination HIV prevention, with the aim of encouraging more effective AIDS responses tailored to the specific needs of MSM at the country level. 

Our study evaluated the impact of social factors such as homophobia, service provider stigma, violence, community engagement, connection to gay community, comfort with service provider, and outness on access to condoms, lubricants, HIV testing, and HIV treatment. We also examined the relationship of these factors to PrEP acceptability. 

We hypothesized a priori that access to condoms, lubricants, HIV testing, and HIV treatment, and PrEP acceptability would be:

1. negatively associated with homophobia, violence toward MSM, violence toward men living with HIV, and service provider stigma;
2. positively associated with community engagement, connection to gay community, comfort with service provider, and being out as gay or MSM; and
3. positively associated with living in a high-income country compared to living in a low, lower-middle or upper-middle income country.

In addition, we hypothesized that PrEP acceptability would be

(4)  positively associated with PrEP knowledge; and
(5)  negatively associated with perceived stigma associated with PrEP.

## 2. Materials and Methods

### 2.1. Recruitment and Implementation

From 23 April to 20 August 2012, we recruited a global convenience sample of MSM to complete the 30-minute online survey. Survey participants were recruited via the MSMGF's networks of community-based organizations focused on advocacy, health, and social services for MSM. The MSMGF sent E-mail blasts advertising the survey to its nearly 3500 online members representing more than 1500 organizations in over 150 countries. Partnering organizations also disseminated information about the survey through their respective regional and global networks, as well as to local MSM through word of mouth. In addition, the MSMGF recruited participants from online social networking sites popular with MSM in Africa, Asia, Europe, and Latin America. Participation in the survey was completely voluntary and anonymous. 

### 2.2. Measures

The MSMGF designed and implemented the multilingual online survey to identify and explore factors that affect access to HIV services for MSM. The survey also evaluated hypothetical acceptability of PrEP consistent with prior intervention acceptability research [15] and explored correlates of acceptability. 

Based on prior literature reviewed, we identified structural, community, and individual-level factors of significant importance to MSM health and hypothesized their mechanism of action (i.e., barrier or facilitator) on access to and acceptability of components of combination HIV prevention. We developed domain categories, adapted validated scales and items to measure these factors, and then tested our hypotheses. Barrier and facilitator variables were measured using multiple-item scales. All scales ranged from 1 to 5 except *service provider stigma*, which ranged from 0 to 1. 

To assess reliability of these scales, we calculated Cronbach alphas overall. Cronbach alphas were also calculated by survey language and by participants' region of residence. As shown in Table 1, overall reliability of scales used in the analyses was acceptable (alpha levels ranged from 0.71 to 0.85). 

The four accessibility outcomes of interest were measured using 5-level variables, with the lowest level indicating complete inaccessibility and the highest level indicating complete accessibility. For analysis, these variables were dichotomized so that respondents were considered to have access if they reported the highest level of accessibility. 

The relationships between PrEP acceptability and hypothesized barriers and facilitators were also examined. PrEP knowledge was measured by asking two yes/no questions about PrEP and assigning a score depending on the respondent's answers to both questions. 

Individual-level sociodemographic information and HIV-related clinical characteristics were collected. These included country of residence (used to determine region of residence and country income), age, sexual orientation, education, housing status, personal income, minority status (i.e., belonging to a racial or ethnic minority group in one's country), time since last HIV test, HIV status, and CD4 count (among survey participants who reported being HIV positive).

Country income was also investigated for its potential impact on access to services and PrEP acceptability. The country income variable was derived from World Bank classifications of country income [16]. 

The survey was originally developed in English and then translated into Chinese, French, Georgian, Russian, and Spanish, then quality-checked by key informants at the country-level utilizing back translation techniques [17]. A final draft of the survey was then pilot tested in English, Spanish, and French with key informants in an effort to increase its face validity among prospective respondents for whom the survey was intended [18].

### 2.3. Data Analysis

For this analysis, we excluded participants with missing or incomplete responses and participants who self-identified as heterosexual or “straight.” We dichotomized our primary outcomes of interests on service access as reporting the highest level of accessibility (i.e., “easily accessible”) versus otherwise. 

Because access to HIV services is partially dependent on contextual variables (e.g., the state of the health system), it is likely that observations within countries are correlated with each other. Similarly, observations of PrEP acceptability might be correlated within countries since detailed information on PrEP might be differentially available across countries.

We used two different approaches for analyzing the data while accounting for this within-country correlation. These approaches were each consistent with the different goals of bivariate and multivariable analyses, respectively [19]. 

In bivariate analysis, we fitted regression models estimated using general estimating equations (GEE) with exchangeable correlation structure. Clusters were defined by country of residence. This approach allowed us to calculate the crude associations between the predictors and outcomes of interest; that is, the approach allowed us to calculate odds ratios that were not adjusted for potential confounders or unmeasured contextual factors. We used Wald tests to determine the statistical significance of predictors. Those variables that were statistically significant (with *P* < 0.2) were included in the multivariable model.

In multivariable analysis, we fitted logistic random effects regression models with random intercepts for respondents' country of residence. These models evaluated the relationship between our independent variables of interest and reporting of the highest level of access to condoms, lubricants, HIV testing, and HIV treatment, while adjusting for potential confounders (age, HIV status, education, housing status, personal income, and minority status) and also controlling for unmeasured contextual variables that occur at the country level. The continuous outcome for PrEP acceptability was modeled using multivariable linear random effects regression. 

All data analysis was carried out using the statistical package, R.

## 3. Results

### 3.1. Respondent Characteristics

Of the 5779 men who accessed the survey, 3748 participants met the criteria for inclusion in this analysis. The majority of surveys were completed in English (58%, *N* = 2190), followed by Spanish (17%, *N* = 654), Russian (12%, *N* = 434), Chinese (9%, *N* = 339), French (3%, *N* = 117), and Georgian (<1%, *N* = 14). The mean age of participants was 35 (range: 12–90 years old). Participants described themselves as “gay” (89%, *N* = 3328) and “bisexual” (11%, *N* = 420). 

A total of 145 countries were represented in the sample analyzed. Twenty-one percent of respondents were from low or lower-middle income countries. The sample contained a high degree of diversity by region. The sample was also diverse in regard to individual-level demographic variables, including age, education level, housing status, and personal income level (see Table 2). 

Fifty-five percent of HIV-negative respondents (*N* = 1709) reported having been tested for HIV in the last 12 months, and 21% indicated having never been tested for HIV. Eighteen percent of respondents reported that they were living with HIV (*N* = 669). Of these respondents, the majority (83%) reported that they were taking antiretroviral medication. Forty-four percent of men living with HIV also reported a CD4 count of 500 or above. Among study participants living with HIV whose CD4 count was lower than 350, 21% reported not taking antiretroviral medications.

**Figure 1:**
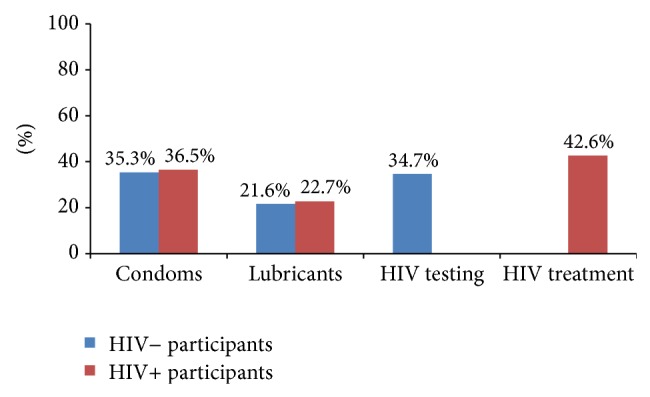
Percent of respondents who reported easy access to services.

A low percentage of respondents reported that condoms, lubricant, and HIV testing were easily accessible (see Figure 1).

Nearly half of HIV-negative respondents reported low levels of knowledge about PrEP (48%), with the remaining participants split between medium (23%) and high (29%) levels of PrEP knowledge.

### 3.2. Bivariate Analyses: Factors Associated with Service Access and PrEP Acceptability

In bivariate analyses, homophobia and experiences of violence for being MSM were significantly associated with lower odds of having easy access to condoms, lubricants, HIV testing, and HIV treatment. Increased service provider stigma was significantly associated with lower odds of having easy access to condoms, lubricants, and HIV testing but was not associated with access to HIV treatment. Among respondents living with HIV, having experienced violence for being HIV positive was significantly associated with lower access to HIV treatment. 

Conversely, community engagement, connection to gay community, and comfort with service provider were each significantly associated with higher odds of having easy access to condoms, lubricants, HIV testing, and HIV treatment. Bivariate associations were also found between country income, age, housing status, minority status, and easy access to HIV service types assessed. The odds ratio for each of these associations is shown in Table 3 below. 

Homophobia (*β* = 0.20; 95% CI: 0.15–0.25, *P* = 0.000), service provider stigma (*β* = 0.18; 95% CI = 0.07–0.30, *P* = 0.002), and having experienced violence for being MSM (*β* = 0.17; 95% CI = 0.12–0.22, *P* = 0.000) were significantly and positively associated with greater PrEP acceptability, whereas outness (*β* = −0.11; 95% CI: −0.13 to −0.08, *P* = 0.000) and community engagement (*β* = −0.09; 95% CI: −0.17 to −0.02, *P* = 0.018) were significantly and negatively associated. PrEP knowledge (*β* = −0.18; 95% CI: −0.23 to −0.14) and PrEP stigma (*β* = −0.42; 95% CI: −0.45 to −0.39, *P* = 0.000) were also negatively associated with PrEP acceptability. Finally, compared to participants in high-income countries, participants expressed higher acceptability of PrEP in low income (*β* = 0.84; 95% CI: 0.59–1.11), lower-middle income (*β* = 0.59; 95% CI: 0.48–0.69), and upper-middle income (*β* = 0.37; 95% CI: 0.29–0.45) countries.

### 3.3. Adjusted Multivariable Analyses

As summarized in Table 4, the highest level of access to condoms was independently associated with lower homophobia, fewer experiences of service provider stigma, more community engagement, greater connection to gay community, more comfort with service provider, and less outness, while adjusting for having experienced violence for being MSM, country income, and demographic variables. 

The highest level of access to lubricants was independently associated with lower homophobia, more community engagement, greater connection to gay community, more comfort with service provider, and less outness, while adjusting for service provider stigma, having experienced violence for being MSM, country income, and demographic characteristics. 

The highest level of access to HIV testing was independently associated with lower homophobia, greater connection to gay community, and more comfort with service provider, while adjusting for provider stigma, outness, having experienced violence for being MSM, country income and demographic characteristics. 

Among participants living with HIV, the highest level of access to HIV treatment was independently associated with lower homophobia and higher comfort with service provider, while adjusting for outness, having experienced violence for being MSM, community engagement, connection to gay community, country income, and demographic characteristics.

Results of the multivariable linear random effects regression model indicate that higher acceptability of PrEP was independently associated with lower PrEP stigma (*β* = −0.51; 95% CI: −0.55 to −0.48, *P* = 0.000), less outness (*β* = −0.15; 95% CI: −0.18 to −0.12, *P* = 0.000), more service provider stigma (*β* = 0.12; 95% CI: 0.02 to 0.23, *P* = 0.021), and less knowledge about PrEP (*β* = −0.14; 95% CI: −0.18 to −0.10, *P* = 0.000), while adjusting for homophobia, having experienced violence for being MSM, community engagement, country income level, and demographic characteristics. In this model, respondents in high-income countries reported less acceptability of PrEP than respondents in low-income (*β* = 0.55; 95% CI: 0.27 to 0.82, *P* = 0.000), lower-middle-income (*β* = 0.43; 95% CI: 0.25 to 0.61, *P* = 0.000), and upper-middle-income countries (*β* = −0.19; 95% CI: 0.02 to 0.35, *P* = 0.031). 

## 4. Discussion

For combination prevention to be successful among MSM, we must ensure access to basic HIV services while promoting acceptability of new biomedical interventions. Our survey findings show that MSM worldwide have unacceptably poor access to the most essential HIV prevention tools. Just more than a third of MSM surveyed reported that condoms and HIV testing were easily accessible, and even fewer (21%) reported easy access to lubricants. Forty-three percent of MSM living with HIV reported that treatment was easily accessible, and these men had significantly less access to condoms or lubricants than they did to HIV treatment. Furthermore, our study found that among MSM homophobia functioned as a consistent barrier to accessing HIV services. We observed that higher levels of homophobia were significantly associated with lower odds of having easy access to condoms, lubricants, HIV testing, and HIV treatment. 

These findings corroborate previous research indicating that structural barriers at the policy and social levels play a central role in hindering access to condoms, lubricants, HIV testing, and HIV treatment for MSM around the world [20–22]. Similarly, in a separate qualitative study of MSM and sexual health conducted by the MSMGF, focus group participants in Kenya, Nigeria, and South Africa described how criminalization and social stigma negatively affected both health seeking behavior and access to services [23]. Criminalization and stigma often lead to social alienation, poor health and mental health outcomes, and further declines in access to services and health seeking behavior among MSM [24–33]. Our findings call attention to the urgency of addressing structural barriers to HIV service access for MSM. Within efforts to implement combination prevention, structural level interventions that combat homophobia should be prioritized.

Our data also revealed facilitators of HIV service access for MSM. Specifically, we found that greater levels of community engagement, connection to gay community, and comfort with service providers were consistently and significantly associated with greater access to condoms, with greater access to condoms, lubricants, HIV testing, and HIV treatment. This finding is in line with other studies which have documented the importance of local community-based organizations as safe spaces for MSM to meet other men like themselves and to receive health services from knowledgeable, nonjudgmental service providers who understand the health needs of MSM from a holistic perspective [34]. Strong relationships with family and community were noted in these reports as facilitators of health and wellbeing, as was the ability to access stable educational and employment opportunities [23]. 

In addition, we found that acceptability of PrEP was independently associated with lower PrEP stigma. However, individuals who exhibited high knowledge of PrEP reported lower acceptability for PrEP. Prior research has shown similar findings among MSM [35, 36]. Other potential barriers to PrEP acceptability among MSM documented in previous research include the intervention's costs, its moderate efficacy, and potential side effects [37, 38]. It is possible that MSM with high PrEP knowledge also have high levels of awareness of these limitations [39, 40]. Thus, MSM with high knowledge of PrEP may be more cautious of the application of this intervention, especially if local and in-country efforts to increase access to more established HIV prevention and treatment interventions have not been fully realized. 

Also contrary to the expectations of the research team, results from the analysis indicated that outness served as a barrier to condom and lubricant access, while controlling for all other variables examined in the survey (including homophobia and connection to the gay community). Furthermore, we learned that respondents who were less out about their sexual orientation and respondents who reported having experienced violence as a result of being MSM were more likely to find PrEP acceptable. This may be due to the fact that outness can result in further stigmatization of MSM in some country contexts, increasing the impact of homophobia [41]. Because homophobia is a barrier to HIV service access, MSM may be concealing their sexual orientation to secure easier access to condoms and lubricants, especially if outness is viewed with disdain by the mainstream public and therefore stigmatizing [42, 43]. Likewise, MSM who have experienced violence or are living in more difficult country contexts may be more open to prevention options that they perceive as less stigmatizing and/or options which they know less about. More research is needed to better understand these associations.

In summary, findings from our study indicate an urgent need to scale up access to basic proven prevention tools and services with the development and rollout of combination prevention for MSM. This is especially critical when considering the interdependent nature of different prevention interventions in maximizing the potential effectiveness of combination approaches. For example, minimizing condom slippage and breakage during anal sex can be aided by proper use of condom-compatible lubricants [44], which is only possible if lubricants are easily accessible. Similarly, the success of PrEP will be contingent upon the success of HIV-testing programs [45]. Challenges accessing any one intervention will likely set off a domino effect, undermining the overall potential of combination prevention approaches for MSM. 

### 4.1. Limitations

This study had several limitations that are important to note. First, the survey data was gathered using a convenience sample, creating the possibility of selection bias for MSM who are more socially connected to MSM organizations or online MSM communication infrastructure, as well as those who have web and E-mail access. As a likely result, levels of participation were limited among MSM in regions where internet access is generally difficult, including Sub-Saharan Africa and the Pacific Islands. On the other hand, levels of participation may have been greater among MSM with higher levels of community involvement with MSM organizations. Hence, our findings may not be generalizable to all MSM. 

It is striking that, among a sample of MSM, most of whom are linked to MSM organizations from which we recruited and to MSM-focused websites where the study was advertised, the proportion of MSM with easy access to condoms, lubricants, testing, and treatment is low. It is possible that among MSM who are not connected with MSM organizations or who do not have access to the internet, access to HIV services is even lower. Moreover, there may also be selection bias for MSM who are particularly motivated to participate. Thus, it is conceivable that data from our sample may be overestimating levels of access and knowledge. 

In addition, our analyses explored the relationship between understudied social and structural factors and access to HIV services, testing multiple *a priori* hypotheses informed by the literature. Given the exploratory nature of these analyses, we did not formally adjust for multiple comparisons; thus, findings of nominal significance should be interpreted with caution [46]. 

Finally, the cross-sectional design of this study limits our ability to make causal inferences from our findings. 

## 5. Conclusion

The study findings underscore the need to improve access to basic HIV prevention and treatment services among MSM before we can fully realize the potential of well-planned, locally relevant combination prevention. Structural, community, and individual-level barriers and facilitators to service access must be addressed on multiple fronts. Interventions must both disrupt the negative effects of barriers *and* support the protective effects of facilitators [47]. Given the positive impact of community engagement and comfort with service providers on access to services, supporting MSM-led community-based organizations to provide a safe space for MSM to access services and connect with the local gay community may be a highly effective strategy for addressing these issues [48]. Finally, when considering the implementation of combination prevention, study findings indicate a need for the dissemination of more and better information about PrEP. Adequately addressing knowledge, perceptions and concerns MSM have about HIV prevention interventions, including but not limited to PrEP, may be critical to their acceptance as part of combination prevention approaches. 

## Figures and Tables

**Table 1:** Reliability scores for survey scales by region.

	Asia	Caribbean	E. Europe and Central Asia	Latin America	Middle East and N. Africa	Oceania	S-Saharan Africa	W N Europe N America	Overall
*Homophobia*: perceptions of homophobia in participant's country (e.g., in your country, how many people believe that a person who is gay/MSM cannot be trusted?)	0.77	0.76	0.69	0.73	0.79	0.81	0.83	0.77	**0.85**

*Provider stigma*: experiences of stigma from health providers (e.g., in your country, has a health provider ever treated you poorly because of your sexuality?)	0.74	0.61	0.71	0.80	0.69	0.66	0.69	0.68	**0.72**

*Violence-MSM*: experiences of violence for being perceived to be MSM (e.g., in the past 12 months, how often were you physically assaulted (slapped, punched, pushed, hit, or beaten) for being gay/MSM?)	0.74	0.81	0.83	0.75	0.84	0.64	0.88	0.64	**0.81**

*Violence-HIV* ∗: experiences of violence for being HIV positive (e.g., in the past 12 months, how often were you physically assaulted (slapped, punched, pushed, hit, or beaten) for being HIV positive?)	0.77	0.79	0.73	0.64	0.85	0.44	0.89	0.53	**0.75**

*Negative consequences for outness*: negative experiences because the participant's sexuality is known to others (e.g., how often have you experienced negative consequences as a result of coworkers knowing that you are attracted to men?)	0.74	0.80	0.68	0.69	0.83	0.65	0.72	0.64	**0.71**

*PrEP stigma* ∗: perceptions of stigma associated with taking PrEP (e.g., if you thought other people would find out that you were taking PrEP drugs to avoid being infected with HIV, how likely is it that you would use PrEP?)	0.79	0.68	0.76	0.74	0.77	0.64	0.73	0.67	**0.74**

*Community engagement*: level of engagement in social activities with other MSM (e.g., during the past 12 months, how often have you participated in gay social groups or in activities such as a book or cooking club?)	0.75	0.78	0.71	0.78	0.59	0.71	0.82	0.76	**0.76**

*Connection to gay community*: the degree to which the participant feels connected to a community of MSM (e.g., how connected do you feel to the gay community where you live?)	0.78	0.75	0.69	0.80	0.69	0.80	0.84	0.79	**0.78**3

*Comfort with provider*: degree of comfort with health provider (e.g., in your country, how comfortable would you feel discussing HIV with your health care provider?)	0.77	0.76	0.73	0.70	0.83	0.72	0.71	0.76	**0.81**

*Outness*: to what degree the participant's sexuality is known to others (e.g., how many of your coworkers know that you are attracted to men?)	0.81	0.86	0.76	0.80	0.83	0.79	0.82	0.76	**0.84**

*PrEP knowledge* ∗: (e.g., do you know what PrEP is? Have you ever heard of taking HIV medications to avoid being infected with HIV?)	0.61	0.60	0.69	0.69	0.85	0.72	0.73	0.69	**0.72**

*PrEP acceptability* ∗: (e.g., how comfortable are you with the idea of using HIV medications to avoid becoming infected with HIV?)	0.78	0.78	0.79	0.77	0.85	0.85	0.84	0.84	**0.82**

^*^The scales for PrEP knowledge, stigma, and acceptability were only measured among respondents who reported being HIV negative or being unsure of their HIV status. Violence-HIV was only measured among respondents who reported living with HIV.

**Table 2:** Respondent sociodemographic and clinical characteristics.

	All participants		Included in analysis	
	*n*	%	*n*	%
Total	**5779**		**3748**	
Region				
Asia	1635	28%	980	26%
Caribbean	126	2%	89	2%
Eastern Europe and Central Asia	966	17%	629	17%
Latin America	880	15%	567	15%
Middle East and North Africa	129	2%	67	2%
Oceania	288	5%	226	6%
Sub-Saharan Africa	380	7%	202	5%
Western and Northern Europe and North America	1375	24%	988	26%
Age category				
<18	72	1%	35	1%
18–25	1221	22%	699	19%
26–30	1194	22%	772	21%
31–40	1426	26%	989	26%
41–50	839	15%	626	17%
51–60	511	9%	414	11%
>60	242	4%	212	6%
Sexual orientation				
Other	81	2%	0	0%
Homosexual/gay	4459	84%	3328	89%
Bisexual	676	13%	420	11%
Heterosexual/straight	102	2%	0	0%
Education				
No postsecondary	1034	19%	652	17%
Postsecondary	4358	81%	3096	83%
Housing status				
Stable place to live	4160	77%	2990	80%
Unstable or no place to live	1232	23%	758	20%
Personal income				
None	561	10%	310	8%
Low/impoverished	433	8%	288	8%
Low middle	1713	32%	1202	32%
Middle	2379	44%	1725	46%
High	306	6%	223	6%
Time since last HIV test (HIV-negative participants)				
<6 months	1195	37%	1142	37%
6–12 months	590	18%	567	18%
1–3 years	482	15%	459	15%
>4 years	282	9%	273	9%
I have never been tested in HIV	679	21%	638	21%
Time since last HIV test (HIV-positive participants)				
In the last 6 months	259	37%	248	37%
Between last 6 months and 1 year ago	56	8%	50	7%
1–3 years ago	71	10%	64	10%
More than 3 years ago	316	45%	306	46%
I have never been tested in HIV	1	0	1	0
HIV status				
HIV negative or status unknown	3228	82%	3079	82%
HIV positive	703	18%	669	18%
CD4 count				
<500	386	56%	373	56%
>500	309	44%	296	44%

**Table 3:** Bivariate associations between hypothesized predictor variables and highest access to HIV services.

	Condoms			Lubricants			HIV testing∗			HIV treatment∗		
	OR	CI	*P*	OR	CI	*P*	OR	CI	*P*	OR	CI	*P*
Homophobia	**0.53**	**0.48–0.59**	**0.000**	**0.39**	**0.35–0.44**	**0.000**	**0.38**	**0.33–0.42**	**0.000**	**0.38**	**0.29–0.49**	**0.000**
Violence-MSM	**0.84**	**0.76–0.93**	**0.001**	**0.67**	**0.58–0.78**	**0.000**	**0.73**	**0.65–0.83**	**0.000**	**0.64**	**0.51–0.80**	**0.000**
Violence-HIV∗										**0.60**	**0.43–0.84**	**0.003**
Provider stigma	**0.57**	**0.46–0.70**	**0.000**	**0.69**	**0.53–0.91**	**0.007**	**0.61**	**0.47–0.80**	**0.000**	0.77	0.54–1.10	0.152
PrEP stigma												
Outness	**1.20**	**1.14–1.26**	**0.000**	**1.22**	**1.15–1.30**	**0.000**	**1.37**	**1.29–1.46**	**0.000**	**1.28**	**1.08–1.51**	**0.005**
Community Engagement	**1.59**	**1.40–1.79**	**0.000**	**1.53**	**1.34–1.75**	**0.000**	**1.72**	**1.49–1.98**	**0.000**	**1.33**	**1.03–1.72**	**0.032**
Connection to gay community	**1.41**	**1.29–1.53**	**0.000**	**1.41**	**1.27–1.56**	**0.000**	**1.54**	**1.40–1.69**	**0.000**	**1.26**	**1.05–1.53**	**0.014**
Comfort with provider	**1.72**	**1.59–1.86**	**0.000**	**1.97**	**1.78–2.19**	**0.000**	**2.40**	**2.17–2.66**	**0.000**	**1.74**	**1.45–2.09**	**0.000**
PrEP knowledge												
Age (measured in decades)	**1.08**	**1.03–1.14**	**0.004**	**1.23**	**1.16–1.30**	**0.000**	**1.41**	**1.32–1.50**	**0.000**	**1.18**	**1.02–1.36**	**0.027**
*Country income *			**0.000**			**0.000**			**0.000**			**0.000**
High income (referent)												
Low income	**0.60**	**0.38–0.93**		**0.15**	**0.07–0.34**		**0.43**	**0.27–0.70**		**0.19**	**0.06–0.63**	
Lower-middle income	**0.55**	**0.45–0.68**		**0.30**	**0.23–0.39**		**0.26**	**0.20–0.32**		**0.38**	**0.21–0.67**	
Upper-middle income	**0.49**	**0.42–0.57**		**0.32**	**0.27–0.39**		**0.29**	**0.24–0.35**		**0.56**	**0.38–0.83**	
*HIV status *			0.565			0.542						
No HIV positive (referent)												
HIV positive	1.05	0.88–1.25		1.07	0.87–1.31							
*Education *			0.514			0.078			0.985			0.381
No postsecondary (referent)												
Postsecondary	0.94	0.79–1.13		0.83	0.67–1.02		1.00	0.82–1.22		0.83	0.55–1.25	
*Personal income *			0.026			0.020			0.000			0.246
None (referent)												
Low income/impoverished	1.22	0.88–1.71		1.17	0.79–1.72		1.40	0.95–2.06		1.13	0.40–3.16	
Low-middle	0.93	0.72–1.21		0.97	0.71–1.31		1.06	0.79–1.41		0.96	0.37–2.44	
Middle	1.19	0.92–1.53		1.24	0.92–1.67		1.32	1.01–1.73		0.83	0.33–2.13	
High	1.24	0.87–1.77		1.54	1.03–2.29		2.00	1.37–2.93		1.93	0.59–6.26	
*Housing status *			**0.000**			**0.000**			**0.000**			0.078
Stable place to live (referent)												
Unstable or no place	**0.67**	**0.56**–**0.80**		**0.60**	**0.47**–**0.75**		**0.56**	**0.46**–**0.69**		0.70	0.47–1.04	
*Minority status *			**0.001**			**0.025**			**0.030**			0.127
Not minority (referent)												
Minority	**1.30**	**1.11**–**1.52**		**1.22**	**1.02**–**1.45**		**1.21**	**1.02**–**1.44**		0.78	0.57–1.07	

^*^Violence-HIV and HIV treatment access were only measured among participants who reported being HIV positive. Bivariate associations with HIV testing access were calculated among HIV negative participants.

**Table 4:** Multivariable logistics random effects modeling of factors associated with access to HIV prevention and treatment services.

	Condoms			Lubricants			HIV testing∗			HIV treatment∗		
	OR	CI	*P*	OR	CI	*P*	OR	CI	*P*	OR	CI	*P*
Homophobia	**0.65**	**0.56–0.75**	**0.000**	**0.54**	**0.46**–**0.65**	**0.000**	**0.64**	**0.54**–**0.76**	**0.000**	**0.52**	**0.36**–**0.76**	**0.001**
Violence-MSM	0.98	0.87–1.12	0.806	0.91	0.77–1.08	0.289	0.90	0.77–1.06	0.200	1.14	0.77–1.67	0.518
Violence-HIV∗										0.71	0.42–1.19	0.191
Provider stigma	**0.72**	**0.57**–**0.91**	**0.007**	1.12	0.84–1.48	0.445	1.14	0.84–1.55	0.408			
Outness	**0.93**	**0.87**–**0.99**	**0.034**	**0.87**	**0.80**–**0.95**	**0.002**	0.99	0.91–1.07	0.719	1.13	0.92–1.38	0.238
Community engagement	**1.26**	**1.09**–**1.47**	**0.002**	**1.25**	**1.04**–**1.48**	**0.014**	1.18	0.98–1.42	0.081	1.14	0.80–1.62	0.465
Connection to gay community	**1.18**	**1.06**–**1.30**	**0.002**	**1.18**	**1.05**–**1.34**	**0.008**	**1.21**	**1.06**–**1.36**	**0.003**	1.10	0.85–1.42	0.483
Comfort with provider	**1.40**	**1.27**–**1.54**	**0.000**	**1.53**	**1.36**–**1.72**	**0.000**	**1.85**	**1.65**–**2.08**	**0.000**	**1.82**	**1.40**–**2.38**	**0.000**

^*^Violence-HIV and HIV treatment access were only measured among participants who reported being HIV positive. The model for HIV testing included only HIV negative participants.
